# Molecular identification of *Mycobacterium bovis* in patients with tuberculosis attending a tertiary care hospital in South India

**DOI:** 10.4314/ahs.v24i3.10

**Published:** 2024-09

**Authors:** Imola Jamir, Noyal Mariya Joseph, Rachana Kannambath, Pradeep Kumar

**Affiliations:** JIPMER, Microbiology

**Keywords:** TB, *M. bovis*, line probe assay

## Abstract

**Background:**

Mycobacterium (M.) bovis is a member of Mycobacterium tuberculosis complex (MTBC). Clinical infection caused by *M. bovis* is indistinguishable from other MTBC and could pose a potential challenge for control of TB epidemic due to its zoonotic nature. Availability of reliable molecular diagnostic methods such as Genotype MTBC based on line probe assay (LPA) paves way for reliable differentiation of M. bovis from other MTBC.

**Objective:**

To determine the proportion of Mycobacterium bovis among the Mycobacterium tuberculosis complex isolates from patients with tuberculosis.

**Methods:**

In our study, we analysed MGIT positive cultures and performed Line probe assay (LPA) for identification of MTBC isolates. Total of 206 patient samples were taken, 104 pulmonary and 102 from extrapulmonary sites.

**Results:**

*M.tuberculosis/M.canettii* was isolated in all pulmonary specimens (100%). Among 102 extrapulmonary samples, 99 % was identified as M.tuberculosis/M.canettii, and 1 % as M. bovis BCG.

**Conclusion:**

Our study suggests that zoonotic TB by *M. bovis* may not be as prevalent in India and hence may not constitute a significant risk to public health in India.

## Introduction

*Mycobacterium bovis* is one of the member of the *Mycobacterium tuberculosis* complex (MTBC) which comprises of Mycobacterium tuberculosis (MTB), *M. africanum, M. canettii, M. bovis, M. bovis BCG, M. microti, M. caprae, M. pinnipedii, M. orygis*. The clinical infection caused by *M. bovis* is indistinguishable from those caused by other members of MTBC. 1 Tuberculosis (TB) caused by the different members of the MTBC is a global problem. The End TB strategy by WHO aims to end the global tuberculosis (TB) epidemic by 2035.^2^ However, *M. bovis* could pose a potential challenge for the control of TB epidemic, because of its zoonotic nature. In the rural areas of India, people often live in close proximity to cows (the natural host of *M. bovis*) which increases the risk of transmission of *M. bovis* from animals to humans. Moreover, M. bovis is intrinsically resistant to pyrazinamide (one of the first line antitubercular drug), which may pose additional challenge. 1 Awareness of the magnitude of problem due to *M. bovis* infection in humans is very important to plan appropriate strategies at the national level for control of TB in India. The incidence of tuberculosis caused by M. bovis in India is unknown or underestimated as the routinely used diagnostic methods are not capable of differentiating *M. bovis* from the other members of MTBC including *M. tuberculosis*. 1 Availability of reliable molecular diagnostic methods such as Genotype MTBC kit based on line probe assay (LPA) paves way for reliable differentiation of *M. bovis* from other members of MTBC. In our study, we analysed MGIT positive cultures and performed line probe assay for identification of MTBC isolates.

## Methods

### Study design and duration

A hospital-based cross-sectional study was performed in Department of Microbiology after approval from the Institutional Ethics Committee. The laboratory confirmed MTBC isolates from pulmonary and extrapulmonary TB patients were included after satisfying the inclusion and exclusion criteria. A total of 206 patient samples were taken, of which 104 was pulmonary and 102 from extrapulmonary sites. All pulmonary and extrapulmonary MTBC isolates were taken consecutively. Demographic patient details such as age, gender and specimen type were noted by a Senior Resident posted in the laboratory and provided in a de-identified manner. Inclusion criteria was MTBC isolates from pulmonary and extrapulmonary TB patients during the study period (January 2020 to December 2021). Exclusion criteria was repeat isolates from the same patient. The sample size based on the expected prevalence of 12.6 % (Bapat PR et al, 2017) 3 and the absolute precision as 4 % and confidence level at 95% was 265. However, due to limited funding, we studied a sample size of 206 MTBC isolates. Pulmonary samples such as sputum, BAL and extrapulmonary samples like CSF, pleural fluid, urine, lymph node aspirate, pus, tissue bits, ascitic fluid are included in this study.

### Sample processing

Collected specimens were either processed immediately or stored at 2-8°C until processed. First, samples underwent decontamination by NALC-NaOH method and the sediments were then inoculated into BACTEC MGIT (Mycobacteria growth indicator tube) 960 and incubated at 37°C. The MGIT tubes flagged as positive were identified as MTBC using rapid detection of MPT64 antigen. All the isolates were then tested using Genotype MTBC line probe assay (LPA) for identification of M. bovis and other members of MTBC.

## Results

### Age and gender distribution

A total of 206 patient samples were included in the study, of which 104 was pulmonary and 102 extrapulmonary samples. Of 104 patients with PTB, 73 (70.2 %) were male and 31 (29.8 %) were female. Mean age of the patients with pulmonary tuberculosis (PTB) was 43.10 ± 16.01 (range, 7 to 75). Of 102 patients with EPTB, 59 (57.8%) were male and 43 (42.2 %) were female. Mean age of the patients with extrapulmonary tuberculosis (EPTB) was 40.19 ± 16.95 (range, 1 to 85). Age distribution of patients with MTBC isolation from Pulmonary and Extra-pulmonary specimens is given in Table 1.

**Table 1:** Age distribution of patients with MTBC isolation from pulmonary and extrapulmonary specimens

	Pulmonary		Extrapulmonary	
	Frequency	Percent (%)	Frequency	Percent (%)
0-20	9	8.7	10	9.8
21-40	34	32.7	46	45.1
41-60	45	43.3	35	34.3
61-80	16	15.4	9	8.8
81-100	-	-	2	2.0
**Total**	**104**	**100.0**	**102**	**100.0**

### MTBC speciation

**Figure 1:**
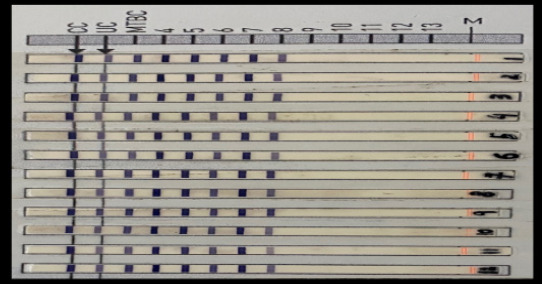
Results of LPA Conjugate control (CC): documents efficiency of conjugate binding and substrate reaction Universal control (UC): positive for all mycobacteria and gram-positive bacteria with high G+C content MTBC: positive for all members of MTBC Bands in 1-8: *M. tuberculosis/M. canettii*

The distribution of MTBC species in pulmonary and extrapulmonary specimens is summarised in Table 2. *M. tuberculosis/M. canettii* was isolated in all the pulmonary specimens (100%). Among the 102 extrapulmonary samples, 99 % (101/102) was identified as *M. tuberculosis/M. canettii*, and 1 % (1/102) as M. bovis BCG from lymph node fine needle aspiration cytology (LN FNAC), Figure 1,2. This LN FNAC sample was from a 5 month-old female child with disseminated BCGiosis.

**Table 2:** Distribution of MTBC in different pulmonary and extrapulmonary specimens

Pulmonary specimens		
	Frequency	Percent ( %)
BAL	5	4.8
Bronchial wash	1	1.0
Endotracheal aspirate	2	1.9
Gastric aspirate	2	1.9
Sputum	94	90.4
**Total**	**104**	**100.0**
**Extrapulmonary specimens**		
	**Frequency**	**Percent ( %)**
Pus	21	20.6
Tissue biopsy	3	2.9
Bone marrow	1	1.0
Bone	1	1.0
CSF	27	26.5
Cyst fluid	1	1.0
Drain fluid	1	1.0
LN FNAC	3	2.9
LN Biopsy	1	1.0
Lung biopsy	2	2.0
Pericardial fluid	1	1.0
Pleural fluid	36	35.3
Urine	4	3.9

**Figure 2:**
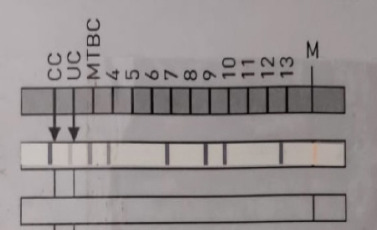
LPA identification of *M bovis* BCG from LN FNAC sample

## Discussion

This cross-sectional study was performed to assess the proportion of *M. bovis* among members of MTBC in laboratory confirmed MTBC isolates from pulmonary and extrapulmonary TB patients. *M. bovis* is zoonotic in nature and there is risk of transmission, especially in rural areas where people live in close proximity to cows. There is no national level surveillance for *M. bovis* and only a few studies have been conducted. Simultaneous control for bovine TB is required to eliminate tuberculosis in humans. The study was done using Genotype MTBC kit based on line probe assay for identification of *M. bovis*.

A total of 206 patient samples were included, of which 104 was pulmonary and 102 from extrapulmonary sites. In our study, the mean age group was 43.10 ± 16.01 and 40.19 ± 16.95 in pulmonary and extrapulmonary TB respectively. The most common age group was 41-60 years (43.3 %) in pulmonary and 21-40 years (45.1%) in extra-pulmonary TB patients. In other Indian studies, the most prevalent age group in pulmonary and extrapulmonary tuberculosis was 20 to 29 years;^4^ in another, 20 to 39 years;^5^ and in a third, 25 to 34 years.^6^ In a study by Sarpal SS et al, mean age of patients was 35.92 ± 15.42 and majority of patients belonged to age group of 25-34 years. 7

In the epidemiology of tuberculosis, age is a critical determinant. TB patients' symptoms, risk of disease progression and treatment results vary with age, most likely due to changes in the immunological response.^8^ The risk of disease following primary TB infection is highest in newborns (under 4 years old) and gradually decreases until it reaches a trough at the age of 5 to 10 years. There is a significant rise in risk during early adulthood and adolescent period (ages 15 to 19 years), with second peak occurring around the age of 20 to 30 years. 9 Disseminated TB, such as miliary TB and TB meningitis are frequent in early infancy. Elderly persons, on the other hand, acquire immunological senescence and those with latent TB infection (LTBI) have a higher chance of developing active TB.^8^,^9^

In our study, out of total 206 patients, 74 (36.0 %) were female and 132 (64.0 %) were male. Of the 104 pulmonary samples, 31 (29.8 %) were female and 73 (70.2 %) were male samples. In the 102 extra-pulmonary samples, 43 (42.2 %) were female and 59 (57.8%) were male samples. This is in concordance with a study from South India where pulmonary samples were from 34 % females and 66 % males; and extra-pulmonary samples from 45 % females and 55 % males. 4 Another study from North India shows similar findings, with 36.1 % female and 63.9 % male prevalence in pulmonary and extra-pulmonary samples. 5 Also, in a study conducted in West Bengal, 30.7 % of TB patients were female and 69.3 % were male.^10^ Various age groups and regions of the world have gender disparities, the cause of which is unknown. The male preponderance for tuberculosis in this study is accordant with data from other nations, and it may indicate work-related, behavioural, or immunological risk factors.

In our research, M. tuberculosis/*M. canettii* was the most prevalent species in pulmonary (100 %) and extrapulmonary (99 %) samples. M. bovis BCG was identified from one LN FNAC sample and no other mycobacteria species were identified. A molecular epidemiological surveillance study conducted in a tertiary care centre in Southern India showed similar findings. In that, of 940 (548 pulmonary and 392 extrapulmonary samples) isolates from positive mycobacteria MGIT tubes, wild-type M. bovis was not identified. The most common MTBC detected was *M. tuberculosis* (97.1 %), followed by M. orygis (0.7%) and M. bovis BCG (0.5%); NTM accounted for 1.6 %. 4 A recent study in China was done on pulmonary samples to identify M. bovis. Similar to our study, no *M. bovis* isolation was found.^11^

Based on earlier studies from various developing countries, it was estimated 2.1% of pulmonary tuberculosis and 9.4% of extrapulmonary tuberculosis was due to M. bovis. 12 Recent estimates suggest about 3.1% of human TB globally are caused by *M. bovis*. In the developing countries, 5–10% of the human TB cases in adults and 30% of pediatric TB cases are due to M. bovis. 13 In the rural areas of UK, where cattle rearing is common, M. bovis was responsible for 5–8.5% of the pulmonary TB in humans, prior to the bovine tuberculosis eradication programme in the UK. 14 Among the MTBC isolates from human TB cases in Mexico, 69% of the extrapulmonary and 31% of the pulmonary isolates were *M. bovis*. 15 In a study conducted in Central India, M. bovis was detected in 12.6% of the blood samples from residents of high TB endemic areas. 16 It also was observed that most of these patients had symptoms suggestive of tuberculosis such as fever, cough with expectoration, night sweats and chest pain. *M. bovis* can cause a clinical presentation similar to that of M. tuberculosis induced TB. 16 In another study conducted among the tribal population of the Melghat region, 2.5% of them were positive for M. bovis and many of them had symptoms consistent with pulmonary tuberculosis.^17^ However, all of these studies relied on blood serology or PCR rather than the culture isolation of M. bovis, which is the definitive proof of *M. bovis* infection.

The threat of zoonotic TB to tuberculosis control is becoming more widely recognised. Evidence is emerging that other members of the MTBC, such as *M. orygis*, may also cause human infections. However, *M. bovis* is still classified as the primary agent of zoonotic tuberculosis by organisations like the WHO.^4^ In a recent South Indian study, 7 cases of TB were found to be caused by M. orygis, in concordance with other reports from South Asia.4 M. orygis has also been found in cattle and rhesus monkeys in Bangladesh. These findings suggest that MTBC complex members, other than *M. bovis*, may be more widespread in cattle in these countries and surveillance studies limited to *M. bovis* may underestimate the incidence of zoonotic TB. Other MTBC subspecies capable of producing human illness should be included in the operational definition of zoonotic TB. In our study, LPA kit did not cover *M. orygis* and hence its prevalence cannot be estimated. However, *M. bovis* was not detected in any of the samples.

The strength of our study is that instead of biochemical assays, a line probe assay (LPA) was utilised for speciation, and it is a quick and accurate diagnostic tool for MTBC speciation. Our research has the following limitations: Due to a lack of financial resources, the sample size is less. Also, the isolates collected may be skewed towards places close to the study site. Hence, to achieve a representative data, more research will have to be conducted in other Indian states.

## Conclusion

In our cross-sectional study, M. tuberculosis/*M. canettii* accounted for 99.5 % of species identified in all samples. M. bovis BCG was identified from one lymph node FNAC sample. In our study, there was no pulmonary or extrapulmonary tuberculosis infection by M. bovis. This, along with another study in Southern India by Duffy et al., 4 suggests that zoonotic TB by *M. bovis* may not be as prevalent in India and hence may not constitute a significant risk to public health in India.
